# Interaction between gestational plane of nutrition and lactation diet composition on lactation performance of Alpine goats of different parities

**DOI:** 10.1038/s41598-023-43450-x

**Published:** 2023-09-27

**Authors:** Luana P. S. Ribeiro, Amlan Kumar Patra, Ryszard Puchala, Roger C. Merkel, Arthur Louis Goetsch

**Affiliations:** American Institute for Goat Research, Langston University, Langston, OK USA

**Keywords:** Metabolism, Reproductive biology

## Abstract

A study was conducted with 48 multiparous and 31 primiparous Alpine goats to determine the effects of different nutritional planes during gestation and lactation on feed intake, body weight, body condition score and mass index, blood constituent concentrations, and milk yield and composition. Two gestation supplement treatments (GS; Moderate versus High) were imposed for approximately 5.5 months and two lactation diets (LD; Moderate vs. High) within each GS were fed for 16 wk. The Moderate GS (14.2% crude protein; CP) was given at 1.125% body weight (BW; dry matter basis) and the High GS (16.2% CP) was consumed ad libitum, with alfalfa hay available free-choice to all animals. Moderate and High LD contained 16.0 and 16.9% CP and 34.7 and 30.4% neutral detergent fiber, respectively. Body weight (77.5 vs. 72.0 kg) and body condition score (BCS; 3.22 vs. 3.04) at 11 days before kidding were greater (*P* < 0.05) for High versus Moderate GS, but BW at kidding (62.6 and 64.9 kg; SEM = 1.32) and 3 days later (60.9 and 63.6 kg for Moderate and High GS, respectively; SEM = 1.32) was similar. Litter size (1.9 and 2.4; SEM = 0.59), kid birth weight (3.72 and 3.59 kg; SEM = 0.097), and litter weight (6.55 and 7.13 kg for Moderate and High GS, respectively; SEM = 0.316) were similar between GS diets. However, kid birth weight (3.44 and 3.87 kg; SEM = 0.096) and litter weight (6.23 and 7.46 kg; SEM = 0.364) were greater (*P* < 0.05) for multiparous versus primiparous goats. Dry matter intake during lactation was greater for Moderate than for High GS (*P* ≤ 0.051) in kg/day, % BW, and g/kg BW^0.75^. However, milk fat (3.81, 4.14, 3.85, and 3.77%; SEM = 0.132) and protein concentrations (2.49, 2.50, 2.47, and 2.49%; SEM = 0.047), and raw (2.22, 2.59, 2.39, and 2.45 kg; SEM = 0.173) and energy yields of milk (6.02, 7.42, 6.51, and 6.63 MJ/day for Moderate GS-Moderate LD, Moderate GS-High LD, High GS-Moderate LD, and High GS-High LD, respectively; SEM = 0.453) were not affected by GS, LD, or their interaction. Dry matter intake, milk and its component yields, and heat energy (MJ/day) were higher (*P* < 0.05) for does than for doelings, but BCS and milk protein and fat concentrations were lower (*P* < 0.05) for does. Blood nonesterified fatty acid concentration was not affected by any diets, but there was interaction (*P* < 0.05) between GS and LD for betahydroxybutyric acid concentration. In conclusion, minor to moderate magnitudes of difference in nutritional planes during gestation and lactation had little effect on reproductive and lactation performance, reflecting the considerable capacity of lactating dairy goats for compensatory changes such as feed intake and tissue mobilization and accretion.

## Introduction

Goats of breeds highly selected for milk yield do not consume feed adequate to support the quantity of milk being produced in early to mid-lactation^1,2^. This is also true for other goat breeds with a litter size of at least two, but the magnitude of nutrient and energy deficits is relatively less than with dairy goat breeds depending of course on factors such as the quality and availability of the diet^3,4^. In fact, goats of dairy breeds, such as Alpine, Saanen, and Toggenburg, could be thought of as being genetically programmed for body tissue mobilization in early lactation regardless of nutritive value of the diet, presumably similar to dairy cattle breeds such as Holstein^5^. Even with a diet very high in nutritive value, at least some tissue will be mobilized because of high potential for milk production^4,6–8^.

As alluded to above, it is known that the diet nutritive value and nutritional planes during gestation affect tissue mobilization for support of milk production in early lactation^6,9^. The other most obvious factor influencing this physiological process is the initial body condition, which is affected by quality and amount of diets^4,6,7^. A greater extent and perhaps longer period of tissue mobilization for support of milk production with a high whole-body energy status are possible^6,7^. This is important not only in relation to milk yield and quality but also as impacting feed requirements for tissue replenishment in the late lactation and(or) the dry period^6,7^. Therefore, diet quality and composition during lactation could influence lactational performance, which may also be further impacted by gestational nutritional status. Furthermore, negative energy balance, excessive tissue mobilization and(or) inadequate replenishment during lactation can adversely affect reproductive performance and health, such as a predisposition to metabolic diseases^3,10,11^. And, similar considerations exist for doelings in their first lactation for continued growth and development^3,8^. The nutritional plane and dietary composition during breeding and gestation may affect reproductive performances^12,13^. Further, nutritional planes and diet composition 3 to 4 wk before parturition may also influence the ruminal microbiome and ruminal fermentation, which subsequently could impact milk production when switched to lactation diets^14–16^. It was hypothesized that different nutritional planes during gestation may influence lactational performance and energy utilization in lactating goats fed different lactation diets. Therefore, the objectives of this experiment were to determine the effects of different nutritional planes during gestation and early to mid-lactation on feed intake, body weight, condition score, and mass index, reproductive performance, blood constituent concentrations, milk yield and composition, and energy utilization of Alpine does and doelings.

## Materials and methods

### Animals, treatments, facility, and experimental design

**Figure 1:**
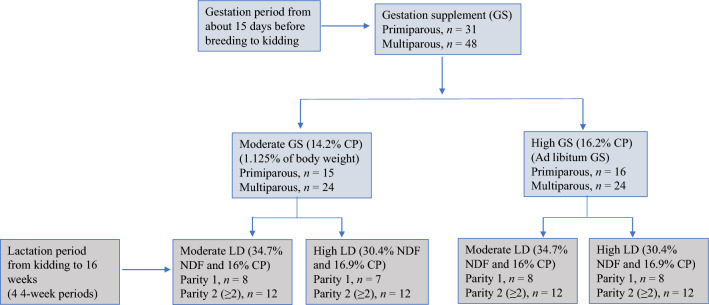
A schematic presentation of the experimental design to investigate the interaction effect of nutritional planes during gestation and diet composition during lactation on lactational performance of Alpine goats. *LD* Lactation diet, *NDF* Neutral detergent fiber, *CP* Crude protein.

The study was conducted according to the guidelines of the AWA and PHS policy and approved by the Langston University Animal Care and Use Committee (Approval Number: 15-119; 15 October 2015). The experimental design was compatible with the ARRIVE (Animal Research: Reporting of In Vivo Experiments) guidelines 2.0. Animals selected from larger groups were 31 Alpine doelings not having previously lactated (i.e., primiparous or parity of 1 when lactating) and 48 does (multiparous; parity of ≥ 2 when lactating), based on factors such as body weight (BW) and anticipated birth date as noted below. Initial BW (mean ± standard error) was 51.4 ± 0.96 and 60.3 ± 1.15 kg and body condition score (BCS, 1 to 5 scale)^17^ was 2.47 ± 0.025 and 2.24 ± 0.030 for doelings and does, respectively. Also, age at kidding was 1.81 ± 0.020 and 4.43 ± 0.225 years for doelings and does, respectively. The treatment arrangement was a 2 × 2 factorial, with two nutritional planes during gestation and two diets fed while lactating (Fig. 1). For addressing gestational performance, animals were randomly assigned to the gestation supplement treatments (GS) for similar BW and BCS within parity with 15–16 doelings and 24 does in each GS treatment. The GS were imposed for approximately 5.5 months when animals were nonlactating, i.e., about 15 days before breeding. Breeding occurred by estrus synchronization and artificial insemination during the first month so that date of birth could be predicted. The GS entailed use of two supplements varying in ingredient composition (Table 1) and level of feeding, which was 1.125% BW on a dry matter (DM) basis for Moderate GS and free-choice (i.e., ad libitum consumption) for High GS. Alfalfa hay was available free-choice for all animals. Diets containing alfalfa hay and supplements had nutrients sufficient to fulfill the energy and protein requirements during gestation. There was one animal group per parity and GS situated in different pastures (i.e., four groups and pastures).

**Table 1:** Gestation supplement and lactation diet composition.

Item^2^	Gestation supplement^1^		Lactation diet	
	Moderate	High	Moderate	High
Ingredient composition (%)				
Alfalfa pellets	0.00	12.50	20.00	20.00
Cottonseed hulls	29.72	18.75	10.00	10.00
Grass hay, coarsely ground	0.00	0.00	20.00	10.00
Wheat middlings	19.60	17.50	10.00	12.86
Rolled oats	20.66	19.25	10.00	12.86
Rolled corn	20.66	19.24	11.55	12.86
Soybean meal	2.40	5.63	11.16	10.99
Soybean oil	1.00	1.88	2.50	3.00
Molasses	3.00	3.13	2.50	5.00
Dicalcium phosphate	1.56	0.00	0.07	0.00
Limestone	0.00	0.88	0.72	0.93
Sodium bicarbonate	0.00	0.31	0.50	0.50
Ammonium sulfate	0.20	0.08	0.20	0.20
Magnesium oxide	0.20	0.13	0.20	0.20
Mineral supplement^3^	1.00	0.63	0.50	0.50
Vitamin supplement^4^	0.10	0.06	0.05	0.05
Trace mineral supplement^5^	0.10	0.06	0.05	0.05
Chemical composition (DM basis)				
Ash (% DM)	8.9 ± 0.35	9.7 ± 0.20	9.0 ± 0.70	9.2 ± 0.39
Crude protein (% DM)	14.2 ± 0.28	16.2 ± 0.20	16.0 ± 0.35	16.9 ± 0.28
Neutral detergent fiber (% DM)	30.6 ± 0.54	30.2 ± 0.68	34.7 ± 1.23	30.4 ± 0.90
Acid detergent fiber (% DM)	17.1 ± 0.58	17.8 ± 0.57	24.4 ± 0.51	21.6 ± 0.70
Gross energy (MJ/kg DM)	17.2 ± 0.08	17.4 ± 0.07	17.9 ± 0.26	18.2 ± 0.11

^1^Moderate = fed at 1.125% body weight (dry matter basis); High = consumed ad libitum.

^2^DM = dry matter.

^3^9–10% Ca, 6% P, 35–40% NaCl, 1% Mg, 1% K, 1% S, 125 mg/kg Co, 150 mg/kg I, 5000 mg/kg Fe, 10 mg/kg Se, 140 mg/kg Zn, 352,000 IU/kg vitamin A, 88,000 IU/kg vitamin D_3_, and 330 IU/kg vitamin E (air-dry basis); Stillwater Milling, Stillwater, OK; Preferred Mineral For Sheep & Goats.

^4^8,800,000 IU/kg vitamin A, 1,760,000 IU/kg vitamin D_3_, and 1100 IU/kg vitamin E; NB-8006, Nutra Blend, Neosho, MO (air-dry basis).

^5^275 mg/kg Co, 2000 mg/kg I, 43,746 mg/kg Fe, 750 mg/kg Se, 18,748 mg/kg Cu, 68,744 mg/kg Zn, and 19,998 mg/kg Mn (air-dry basis).

For the lactation phase, the treatment arrangement was a 2 × 2 factorial, with two GS and two lactation diet treatments (LD). Animals were assigned to two LD treatments (Table 1) with 15–16 doelings and 24 does per LD treatment (7–8 doelings and 12 does per LD treatment within each GS treatment) in three sets of animals kidding at different times every 2 wk, for an average days-in-milk of 3.3 ± 0.21. The allocation was based on BW and BCS within parity and GS. The composition of LD is shown in Table 1, also designated as Moderate and High. The lactation phase of the experiment was 16 wk in length, consisting of four 4-wk periods. The confinement facility where animals were housed during lactation, described by Patra et al.^18^, had 5.57 m × 3.06 m pens. An area of 5.57 m × 1.33 m at the front of each pen had an elevated expanded metal floor, with a flush manure system used once daily. Diets were offered free-choice (120% of consumption on the preceding few days) at 08:00 h before measuring orts in Calan gate feeders (American Calan Inc., Northwood, NH, USA) for individual feeding and recording. Feed intake was calculated subtracting orts from feed offered in each day. Ambient temperature and relative humidity were determined every 30 min with two Hobo® Temperature/RH Data Loggers (model number U12-011; Onset Computer Corp., Bourne, MA, USA) placed in different areas of the facility. A temperature-humidity index was calculated as described by Amundson et al.^19^.

### Measures

During gestation, BW using digital portable weighing balance, average daily gain (ADG), and BCS were measured at 11 ± 0.09 days before kidding, with the same three individuals assessing BCS in a 1 to 5 scale^17^. Linear measures also were determined at this time, which included height at the withers (Wither), length from the point of the shoulder to the hook bone (Hook) and pin bone (Pin), and circumference from heart girth (Heart). Four of the 13 body mass indexes (BMI) described by Liu et al.^20^ were estimated, as noted below.

$$\begin{aligned} & {\text{BMI-WH}} = {\text{BW}}/\left( {{\text{Wither}} \times {\text{Hook}}} \right)\;\left[ {{\text{g}}/{\text{cm}}^{2} } \right] \\ & {\text{BMI-WP}} = {\text{BW}}/\left( {{\text{Wither}} \times {\text{Pin}}} \right)\;\left[ {{\text{g}}/{\text{cm}}^{2} } \right] \\ & {\text{BMI-GH}} = {\text{BW}}/\left( {{\text{Heart}} \times {\text{Hook}}} \right)\;\left[ {{\text{g}}/{\text{cm}}^{2} } \right] \\ & {\text{BMI-GP}} = {\text{BW}}/\left( {{\text{Heart}} \times {\text{Pin}}} \right)\;\left[ {{\text{g}}/{\text{cm}}^{2} } \right] \\ \end{aligned}$$

During lactation, milk yield was determined every day at 07:00 and 1600 h in automatic milking machines (Bou-Matic, DEC International, Madison, WI, USA) connected to a computerized system (Westfalia Systemat, Elk Grove Village, IL, USA) for milk production recording of each doe. Milk samples collected every 2 wk at both times were analyzed separately for fat, protein, and lactose at the certified Dairy Herd Information Laboratory for Goats at Langston University with a MilkoScan 400 analyzer (Foss Electric, Hillerød, Denmark). Somatic cell count (SCC) was determined with a Fossomatic 5000 analyzer (Foss Electric). Milk energy concentration was determined with the equation of NRC^11^ based on concentrations of fat, protein, and lactose. Milk yield and composition data were averaged over 4-wk periods for 16 wk. Body weight, BCS, linear measures, and BMI were determined at the beginning of the lactation period and end of each 4-wk period.

The gestation supplements and lactation diets were sampled weekly and composites were formed for 4-wk periods. Samples were dried at 55º C in a forced-air oven, ground to pass a 1-mm screen, and analyzed for DM, ash^21^, CP (nitrogen × 6.25; Leco TruMac CN, St. Joseph, MI, USA), neutral detergent fiber (NDF) with use of heat stable amylase and containing residual ash^22^ (filter bag technique of ANKOM Technology Corp., Fairport, NY, USA), acid detergent fiber (ADF)^22^, and gross energy^21^ using a bomb calorimeter (Parr 6300; Parr Instrument Co., Inc., Moline, IL, USA).

Heat energy (HE) was determined by use of heart rate (HR) for prediction, as described by Puchala et al.^23,24^. During the last week of each period, HR was recorded for 48 h with six animals per pen, three of each parity, fitted with 10 cm × 10 cm electrodes prepared from stretch conductive fabric (Less EMF, Albany, NY, USA), glued to Vermed PerformancePlus ECG electrodes (Bellows Falls, VT, USA) and attached to the chest just behind and slightly below the left elbow and behind the shoulder blade on the right side. Electrodes were connected by ECG snap leads (Bioconnect, San Diego, CA, USA) to T61 coded transmitters (Polar, Lake Success, NY, USA). Human S610 heart rate (Polar) monitors with wireless connection to the transmitters were used to collect HR data at a 1-min interval. Heart rate data were analyzed using Polar Precision Performance SW software.

The ratio of HE to HR was determined with the same animals used for HR measures while in group pens for 24 h in periods 2 and 3, following 1 day of adaptation to housing in an adjacent room, in four metabolism crates fitted with head-boxes of an indirect, open-circuit respiration calorimetry system (Sable Systems International, North Las Vegas, NV, USA). The LD treatments continued to be imposed during this period. Measures were similar to those in previous studies^23,24^. Oxygen concentration was measured using a fuel cell FC-1B O_2_ analyzer (Sable Systems International), and CH_4_ and CO_2_ concentrations were measured with infrared analyzers (CA-1B for CO_2_ and MA-1 for CH_4_; Sable Systems International). Prior to gas exchange measurements, analyzers were calibrated with gases of known concentrations. Ethanol combustion tests were performed to ensure complete recovery of O_2_ and CO_2_ produced with the same flow rates as used during measurements. Heat energy was determined according to the Brouwer^25^ equation without consideration of urinary nitrogen, and was expressed relative to kg BW^0.75^ during the measurement period. Heart rate measured during the periods was multiplied by the ratio of HE to HR to estimate HE, with HE in MJ/day based on average kg BW^0.75^ in the 4-wk periods.

Blood samples were collected via jugular venipuncture in wk 5, 10, and 15 (i.e., periods 2, 3, and 4) during lactation in the morning at approximately 3 h after feeding into two tubes. One tube contained sodium fluoride and potassium oxalate. Plasma and serum were harvested after centrifuging at 3,000 × *g* and 10º C for 20 min and stored at -20º C. Plasma was analyzed for glucose and lactate with a YSI 2300 Plus Glucose & Lactate Analyzer (YSI Inc., Yellow Springs, OH, USA). Serum was analyzed for nonesterified fatty acids (NEFA) with a Vet Axcel® Chemistry Analyzer (Alfa Wassermann Diagnostic Technologies, West Caldwell, NJ, USA) according to the manufacturer’s instructions and for β-hydroxybutyrate (BHB) by the EnzyChrom™ Ketone Body Assay Kit (EABD-100) of BioAsssay Systems (Hayward, CA, USA).

### Statistical analyses

Most data except litter size were analyzed by mixed effects models with the Statistical Analysis System^26,27^. Fixed effects for measures during gestation were GS, parity, and GS × parity, with the random effect of animal within GS × parity. The GLIMMIX procedure of SAS was used for the categorical variable of litter size. For measures during the lactation phase, 4-wk period was a repeated measure, with the subject of animal within GS × parity × LD. Different covariance structures were compared via Akaike’s Information Criterion, but values were usually lower for Variance Components. Fixed effects were GS, LD, parity, period, and all interactions. Because overall effects were of interest regardless of the significance of interactions involving period, for some variables main effect means are presented along with interaction means. Means were separated by the least significant difference when the treatment F-test was significant (*P* < 0.05). Pearson correlation coefficients between BW, BCS, BMI, ADG, DM intake and milk energy yield (NE_l_) were determined using SAS^27^.

## Results

### Feed composition and temperature and humidity

The ingredient and chemical composition of the gestation phase supplements were not markedly different (Table 1). The only constituent with a moderate difference in concentration was a CP concentration 2 percentage units greater for High GS versus Moderate GS. The primary means by which the two GS treatments might be expected to affect performance differently is their level of GS feeding (1.125% of BW versus ad libitum). Alfalfa hay was available free-choice so that the performance of Moderate GS animals would not be severely restricted, in accordance with typical production practices on many commercial dairy goat farms.

The main differences in ingredients of two LD were a lower level of grass hay and higher levels of wheat middlings, rolled oats, rolled corn, soybean oil, and molasses in the High LD than in the Moderate LD. The concentration of CP was 0.9 percentage units higher in High LD, and levels of NDF and ADF were greater for Moderate LD than for High LD (i.e., 4.3 and 2.8 percentage units, respectively). As for the GS, the Moderate LD were not formulated to greatly restrict performance, again with the intent to simulate common and practical farm settings.

Temperature (12.9 to 27.8 ºC) and the temperature-humidity index (55.8 to 77.7) increased as the lactation period progressed, without marked change in the relative humidity (Table 2). The animals were not subjected to heat or cold stress conditions as discussed by Silanikove and Koluman^28^.

**Table 2:** Average daily temperature (T), relative humidity (RH), and temperature-humidity index (THI) in the confinement facility during the lactation period.

Season	Month	Item^1^	Mean	SEM	Minimum	Maximum
Winter	February	Temperature (°C)	12.9	0.35	6.36	21.1
		RH (%)	60.6	0.70	36.9	76.7
		THI	55.8	0.50	46.3	67.2
Spring	March	Temperature (°C)	17.3	0.12	7.6	25.5
		RH (%)	50.4	0.42	24.0	79.3
		THI	61.7	0.16	49.3	72.4
	April	Temperature (°C)	21.6	0.05	9.4	30.7
		RH (%)	65.4	0.15	33.8	81.4
		THI	68.4	0.07	50.5	81.9
	May	Temperature (°C)	26.1	0.05	20.4	32.4
		RH (%)	66.5	0.12	43.0	79.0
		THI	75.1	0.07	66.8	83.7
Summer	June	Temperature (°C)	27.8	0.07	24.8	31.6
		RH (%)	69.2	0.26	49.5	79.3
		THI	77.7	0.08	74.0	81.4

^1^THI = (0.8 × T) + [(RH/100) × (T − 14.4)] + 46.4 (Amundson et al.^19^).

### Measures in late gestation, at kidding, and start of lactation

At 11 days before birth, BW and BCS were greater (*P* < 0.05) for High GS than for Moderate GS, with magnitudes of difference of 6–8% (Table 3; i.e., 5.5 kg BW and 0.18 BCS units, respectively). Likewise, all BMI were greater for High GS versus Moderate GS. However, doe BW at birth and on day 1 of the lactation phase was not different between GS (*P* > 0.05). Similarly, BMI on day 1 were similar between GS treatments (*P* > 0.05). The GS supplement treatment did not affect reproduction measures of litter size, kid birth weight, or litter weight (*P* > 0.05).

**Table 3:** Effects of gestation supplement treatment and parity on conditions of Alpine goats near the end of gestation and start of the lactation phase.

Item^2^	Source of variation^1^			GS		SEM	Parity		
	GS	PY	GS*PY	Moderate	High		1	2	SEM
11 days before birth									
BW (kg)	0.009	< 0.001	0.381	72.0^a^	77.5^b^	1.44	68.7^a^	80.7^b^	1.45
BCS	0.001	0.044	0.289	3.04^a^	3.22^b^	0.037	3.18^b^	3.08^a^	0.037
BMI-									
WH	0.002	< 0.001	0.073	16.4^a^	17.7^b^	0.29	16.0^a^	18.1^b^	0.29
WP	0.002	< 0.001	0.127	13.0^a^	14.0^b^	0.22	12.7^a^	14.4^b^	0.22
GH	0.011	< 0.001	0.103	12.5^a^	13.1^b^	0.02	12.3^a^	13.3^b^	0.177
GP	0.022	< 0.001	0.219	9.93^a^	10.39^b^	0.140	9.73^a^	10.59^b^	0.139
Day of birth									
BW (kg)	0.224	< 0.001	0.610	62.6	64.9	1.32	58.4^a^	69.1^b^	1.32
Litter size	0.541	0.521	0.752	1.87	2.39	0.591	1.86	2.41	0.582
Kid weight (kg)	0.367	0.002	0.875	3.72	3.59	0.097	3.44^a^	3.87^b^	0.096
Litter weight (kg)	0.265	0.021	0.637	6.55	7.13	0.366	6.23^a^	7.45^b^	0.364
Decrease in BW (kg)									
Total	0.001	0.152	0.375	9.4^a^	12.6^b^	0.67	10.3	11.7	0.67
Total minus litter weight	0.001	0.832	0.420	2.8^a^	5.4^b^	0.53	4.1	4.2	0.53
Day 1									
BW (kg)	0.146	< 0.001	0.826	60.9	63.6	1.32	57.5^a^	66.9^b^	1.32
BCS	0.915	0.351	0.935	2.98	2.98	0.043	3.01	2.95	0.043
BMI-									
WH	0.923	< 0.001	0.452	14.7	14.7	0.32	13.8^a^	15.6^b^	0.31
WP	0.746	< 0.001	0.426	11.9	12.0	0.20	11.2^a^	12.6^b^	0.20
GH	0.284	0.001	0.357	11.3	11.0	0.20	10.7^a^	11.7^b^	0.20
GP	0.402	< 0.001	0.321	9.12	8.97	0.125	8.70^a^	9.39^b^	0.125

^1^GS = gestation supplement treatment; PY = parity (1 or 2 =  ≥ 2).

^2^Day 1 = start of study at an average of 3 days after birth; decrease in BW = difference between values at 11 days before and at birth; BCS = body condition score (1–5); BMI = body mass index; Wither = height at withers; Hook = point of the shoulder to hook bone; Pin = point of the shoulder to pin bone; Heart = heart girth; BMI-WH = BW/(Wither × Hook) [g/cm^2^]; BMI-WP = BW/(Wither × Pin) [g/cm^2^]; BMI-GH = BW/(Heart × Hook) [g/cm^2^]; BMI-WP = BW/(Heart × Pin) [g/cm^2^].

^a,b^Means in a row within grouping without a common superscript letter differ (*P* < 0.05).

Body weight was greater (*P* < 0.05) for multiparous than for primiparous goats at 11 days before kidding, on the day of birth, and at the start of the lactation phase (Table 3). Body condition score was less (*P* < 0.05) for multiparous than for primiparous animals before birth but not different (*P* > 0.05) at the start of the lactation phase. The four BMI on day 1 were greater (*P* < 0.05) for multiparous than for primiparous goats. Litter size was numerically (*P* > 0.05) greater for multiparous versus primiparous goats (i.e., 0.52 kids). This numerically higher litter size coupled with greater (*P* < 0.05) kid birth weight for multiparous goats resulted in greater litter birth weight (*P* < 0.05), with a difference of 1.22 kg.

In addition to evaluation of BW at the times of 11 days before and at birth, differences were considered (Table 3). The degree to which BW was less at birth than earlier was affected by GS (*P* = 0.001) but not parity or their interaction (*P* > 0.05), as was also true for that attributable to tissue and fluid other than associated with fetuses (i.e., total decrease minus litter weight; *P* = 0.001).

### Feed intake, BW, ADG, and blood constituent levels during lactation

Dry matter intake was not influenced by LD (*P* > 0.05), although there were effects of GS on DM intake (Tables 4, 5). Dry matter intake was greater for Moderate GS than for High GS in g/day (*P* = 0.051), % BW (*P* = 0.042), and g/kg BW^0.75^ (*P* = 0.034). Dry matter intake in g/day was influenced by period and parity (*P* < 0.05), although there was an interaction between these factors for each expression of DM intake (*P* < 0.05). Magnitudes of difference relative to BW were not substantial or consistent among periods. Although, it would appear that DM intake by multiparous animals rose faster with advancing time and peaked earlier than that by primiparous animals.

**Table 4:** *P* values for effects of gestation supplement treatment, lactation diet, and parity on BW, ADG, DM intake, and blood constituent concentrations of Alpine goats during the four 4-wk periods.

Source of variation^2^	Variable^1^								
	DMI (g/day)	DMI (% BW)	DMI (g/kg BW^0.75^)	BW (kg)	ADG (g)	GLC (mg/dl)	LAC (mg/dl)	BHB (mmol/l)	NEFA (meq/l)
GS	0.051	0.042	0.034	0.673	0.129	0.572	0.778	0.875	0.475
LD	0.110	0.124	0.106	0.923	0.531	0.190	0.680	0.793	0.413
GS*LD	0.245	0.337	0.283	0.856	0.697	0.146	0.154	0.041	0.230
PY	0.002	0.862	0.364	< 0.001	0.028	0.976	0.004	0.059	0.084
GS*PY	0.821	0.924	0.986	0.655	0.493	0.163	0.385	0.686	0.161
LD*PY	0.217	0.074	0.085	0.539	0.751	0.826	0.635	0.890	0.218
GS*LD*PY	0.910	0.861	0.897	0.577	0.126	0.270	0.226	0.038	0.877
PD	< 0.001	< 0.001	< 0.001	< 0.001	< 0.001	0.002	< 0.001	0.705	0.008
GS*PD	0.432	0.081	0.136	0.118	0.001	0.578	0.780	0.388	0.541
LD*PD	0.444	0.198	0.246	0.104	0.200	0.424	0.582	0.977	0.332
GS*LD*PD	0.835	0.828	0.834	0.798	0.337	0.682	0.013	0.008	0.055
PY*PD	< 0.001	0.002	0.001	< 0.001	0.053	0.136	0.006	0.007	0.154
GS*PY*PD	0.657	0.678	0.676	0.692	0.988	0.901	0.397	0.774	0.427
LD*PY*PD	0.492	0.587	0.556	0.721	0.862	0.840	0.665	0.925	0.210
GS*LD*PY*PD	0.983	0.989	0.991	0.562	0.342	0.109	0.773	0.495	0.387

^1^DMI = DM intake; BW = body weight; ADG = average daily gain; GLC = glucose; LAC = lactate; BHB = β-hydroxybutyrate; NEFA = nonesterified fatty acids. ^2^GS = gestation supplement treatment; LD = lactation diet treatment; PY = parity (1 or 2 =  ≥ 2); PD = period.

**Table 5:** Effects of gestation supplement treatment, lactation diet, and parity on BW, ADG, DM intake, and blood constituent concentrations of Alpine goats during the four 4-wk periods^1,*^.

Item^2^	PY	GS	PD	Moderate GS		High GS		SEM	Period				SEM	Parity		SEM
				Mod LD	High LD	Mod LD	High LD		1	2	3	4		1	2	
DMI (g/day)				2155	2401	2084	2123	88.0	1947^a^	2310^c^	2334^c^	2173^b^	51.0	2052^a^	2330^b^	61.9
	1								1743^a^	2102^b^	2231^c^	2131^bc^	71.7			
	2								2151^bc^	2517^d^	2436^d^	2215^bc^				
DMI (% BW)				3.67	4.07	3.49	3.59	0.158	3.23^a^	3.89^c^	3.97^c^	3.72^b^	0.089	3.69	3.72	0.111
	1								3.13^a^	3.79^bc^	4.01^d^	3.84^bcd^	0.125			
	2								3.34^a^	3.99^cd^	3.94^cd^	3.61^b^				
DMI (g/kg BW^0.75^)				101	113	97	99	4.1	90^a^	108^c^	110^cd^	103^cd^	2.4	101	105	2.9
	1								85^a^	103^cd^	109^d^	105^bc^	3.3			
	2								95^b^	112^d^	110^cd^	101^b^				
BW (kg)				59.3	59.4	60.3	59.8	1.66	60.7^b^	59.7^a^	59.2^a^	59.1^a^	0.85	56.1^a^	63.3^b^	1.17
	1								56.4^a^	55.7^a^	56.1^a^	56.1^a^	1.20			
	2								65.1^d^	63.7^c^	62.4^b^	62.1^b^				
ADG (g)				− 17	− 14	− 42	− 29	12.9	− 75^a^	− 35^b^	5^c^	3^c^	12.9	− 11^b^	− 40^a^	9.1
		Mod							− 37^b^	− 44^b^	38^d^	− 19^bc^	18.2			
		High							− 113^a^	− 26^bc^	− 27^bc^	24^cd^				
Blood constituents^3^																
Glucose (mg/dl)				51.0	50.7	49.4	54.2	1.72		46.9^a^	54.3^b^	52.8^b^	1.49	51.3	51.3	1.21
Lactate (mg/dl)				5.48	6.01	6.37	5.42	0.513		8.84^b^	4.12^a^	4.49^a^	0.435	6.57^b^	5.06^a^	0.361
	1									10.7^c^	4.12^a^	4.88^a^	0.612			
	2									6.96^b^	4.13^a^	4.10^a^				
BHB (mmol/l)				0.45^a^	0.63^ab^	0.67^b^	0.44^a^	0.087								
	1			0.45^a^	0.43^a^	0.52^ab^	0.46^a^	0.121								
	2			0.45^a^	0.83^b^	0.82^b^	0.42^a^									
			2	0.32^a^	0.83^bc^	0.90^c^	0.31^a^	0.127								
			3	0.55^abc^	0.57^abc^	0.49^abc^	0.46^ab^									
			4	0.48^abc^	0.50^abc^	0.63^abc^	0.55^abc^									
NEFA (meq/l)				0.34	0.35	0.38	0.34	0.023		0.37^b^	0.31^a^	0.37^b^	0.018	0.33	0.37	0.016

^1^GS = gestation supplement treatment; LD = lactation diet treatment; Mod = moderate; PY = parity (1 or 2 =  ≥ 2); PD = period.

^2^DMI = DM intake; BW = body weight; ADG = average daily gain; BHB = β-hydroxybutyrate; NEFA = nonesterified fatty acids.

^3^Blood samples were collected in periods 2, 3, and 4.

*Mean values of main effects are presented when there were no significant interaction effects (*P* > 0.05) involving the main effects. Otherwise, data are presented showing interaction effect (*P* < 0.05) or significant main effects (*P* < 0.05).

Body weight was not influenced by GS or LD (*P* > 0.05), was affected by period and parity (*P* < 0.05), and there was a period × parity interaction (*P* < 0.05; Tables 4, 5). The interaction related to similar values for primiparous animals among periods and a ranking (*P* < 0.05) of period 1 > 2 > 3 and 4 for multiparous animals. Neither GS nor LD affected ADG (*P* > 0.05), but there were effects of period, parity, and GS × period (*P* < 0.05), and there was a tendency for a period × parity interaction (*P* = 0.053). Overall, ADG ranked period 1 < 2 < 3 and 4 and average BW loss was greater for parity 2 versus 1 (*P* < 0.05). The primary factor responsible for the period × parity interaction tendency was considerable BW loss by multiparous animals in period 1 relative to multiparous animals in other periods and primiparous goats (i.e., -53, -25, 24, and -9 g for doelings and -98, -45, -33, and 14 g for does in period 1, 2, 3, and 4, respectively; SEM = 18.2). Somewhat similarly, the GS × period interaction was primarily a result of relatively large BW loss in period 1 by High GS animals.

The only factor influencing the concentration of glucose was period, with a lower value in period 2 than in periods 3 and 4 (*P* < 0.05; Tables 4, 5). The concentration of lactate was greatest among periods in period 2 (*P* < 0.05) and greater for primiparous than for multiparous goats (*P* < 0.05). The concentration of BHB was influenced by two and three-way interaction factors. For the two-way interaction between GS and LD, the level was greater for High GS-Moderate LD than for Moderate GS-Moderate LD and High GS-High LD (*P* < 0.05). There were three-way interactions between GS, LD, and parity and GS, LD, and period (*P* < 0.05). The interactions were mainly due to relatively high values for Moderate GS-High LD and High GS-Moderate LD for multiparous but not primiparous goats and in period 2 but not periods 3 or 4. The only factor influencing the NEFA concentration was period, with a lower value for period 3 versus 2 and 4 (*P* < 0.05).

### Milk composition and yield

Milk fat concentration was not influenced by GS or LD (*P* > 0.05), ranked period 1 > 2 > 3 and 4 (*P* < 0.05), and was greater for primiparous versus multiparous goats (*P* = 0.011; Tables 6, 7). However, there was an interaction involving GS, LD, and period (*P* = 0.035), which appeared due largely to relatively high values in period 1 for High LD treatments and a low mean in period 2 for High GS-High LD. The protein concentration in milk also was not affected by GS or LD (*P* > 0.05) but varied among periods and between parities as did the milk fat concentration. There was an interaction between period and parity as well (*P* = 0.030), which related to a greater value in period 2 for primiparous versus multiparous goats and lower levels for both parities in period 2 than in periods 1, 3, and 4. There was an interaction in lactose concentration between period and parity (*P* = 0.011), the nature of which was similar to that in protein concentration. In partial accordance with concentrations of individual constituents, the level of total solids was not affected by GS or LD, ranked (*P* < 0.05) period 1 > 2 and 4 > 3 (*P* < 0.05), and was greater for primiparous than for multiparous animals (*P* = 0.004). However, again, there was an interaction between period and parity (*P* = 0.042), partially attributable to the greatest interaction mean for primiparous in period 1 (*P* < 0.05). Likewise, there was a three-way interaction involving GS, LD, and period (*P* = 0.031), with higher interaction means in period 1 for Moderate GS-High LD and High GS-High LD than for other treatments (*P* < 0.05) except High GS-Moderate LD. The milk energy concentration was similar among GS × LD treatments (*P* > 0.05) and ranked period 1 > 2 > 4 > 3 (*P* < 0.05). There was a three-way GS × LD × period interaction as well (*P* = 0.026). No factors influenced SCC (*P* > 0.05).

**Table 6:** *P* values for effects of gestation supplement treatment, lactation diet, and parity on milk yield and composition of Alpine goats during four 4-wk periods.

Source of variation^3^	Concentration^1^						Yield^2^						
	Fat	Protein	Lactose	TS	GE	SCC	Raw	Fat	Protein	Lactose	TS	GE	GE:DMI
GS	0.207	0.695	0.853	0.354	0.257	0.299	0.956	0.552	0.933	0.928	0.859	0.742	0.990
LD	0.362	0.776	0.054	0.160	0.279	0.122	0.222	0.087	0.131	0.138	0.104	0.098	0.100
GS*LD	0.116	0.897	0.930	0.262	0.180	0.179	0.368	0.093	0.362	0.334	0.218	0.166	0.423
PY	0.011	0.028	0.086	0.004	0.006	0.765	< 0.001	0.006	< 0.001	< 0.001	0.001	0.001	0.332
GS*PY	0.102	0.736	0.700	0.269	0.156	0.620	0.518	0.973	0.459	0.475	0.614	0.724	0.311
LD*PY	0.537	0.479	0.225	0.304	0.416	0.870	0.881	0.733	0.750	0.793	0.755	0.745	0.321
GS*LD*PY	0.062	0.321	0.758	0.092	0.068	0.678	0.946	0.435	0.660	0.908	0.674	0.576	0.645
PD	< 0.001	< 0.001	< 0.001	< 0.001	< 0.001	0.697	< 0.001	< 0.001	< 0.001	< 0.001	< 0.001	< 0.001	< 0.001
GS*PD	0.782	0.252	0.221	0.381	0.525	0.910	0.558	0.969	0.669	0.845	0.871	0.920	0.639
LD*PD	0.084	0.057	0.529	0.088	0.059	0.827	0.317	0.539	0.204	0.517	0.526	0.505	0.697
GS*LD*PD	0.035	0.263	0.097	0.031	0.026	0.052	0.582	0.040	0.355	0.384	0.120	0.067	0.805
PY*PD	0.438	0.030	0.011	0.042	0.121	0.817	< 0.001	0.001	0.005	0.002	0.001	< 0.001	0.272
GS*PY*PD	0.954	0.914	0.717	0.880	0.921	0.610	0.842	0.917	0.998	0.965	0.958	0.939	0.432
LD*PY*PD	0.155	0.382	0.429	0.167	0.132	0.714	0.859	0.219	0.611	0.579	0.313	0.245	0.155
GS*LD*PY*PD	0.293	0.415	0.714	0.567	0.442	0.457	0.388	0.174	0.913	0.703	0.505	0.339	0.586

^1^TS = total solids; GE = gross energy (MJ/kg); SCC = log somatic cell count; % fat, protein, lactose, and TS.

^2^Raw, fat, protein, lactose, and TS (g/day); GE (MJ/day); GE:DMI = GE/dry matter intake (MJ/kg).

^3^GS = gestation supplement treatment; LD = lactation diet treatment; PY = parity (1 or 2 =  ≥ 2); PD = period.

**Table 7:** Effects of gestation supplement treatment, lactation diet, and parity on milk yield and composition of Alpine goats during four 4-wk periods^1,*^.

Item^2^	PY	PD	Moderate GS		High GS		SEM	Period				SEM	Parity		SEM
			Mod LD	High LD	Mod LD	High LD		1	2	3	4		1	2	
Concentration															
Fat (%)			3.81	4.14	3.85	3.77	0.132	4.78^c^	3.91^b^	3.39^a^	3.49^a^	0.103	4.06^b^	3.72^a^	0.093
		1	4.54^de^	5.04^e^	4.61^de^	4.93^e^	0.205								
		2	3.93^bc^	4.16^cd^	4.13^cd^	3.41^ab^									
		3	3.49^ab^	3.45^ab^	3.16^a^	3.47^ab^									
		4	3.29^a^	3.92^bc^	3.51^ab^	3.25^a^									
Protein (%)			2.49	2.50	2.47	2.49	0.047	2.86^c^	2.40^b^	2.21^a^	2.48^b^	0.043	2.54^b^	2.43^a^	0.033
	1							2.94^c^	2.54^b^	2.17^a^	2.51^b^	0.061			
	2							2.78^c^	2.26^a^	2.24^a^	2.45^b^				
Lactose (%)			4.17	4.26	4.18	4.26	0.046	4.33^c^	4.19^ab^	4.07^a^	4.28^bc^	0.046	4.26	4.18	0.032
	1							4.40^c^	4.33^c^	4.00^a^	4.30^bc^	0.065			
	2							4.25^bc^	4.05^a^	4.14^ab^	4.27^bc^				
TS (%)			11.2	11.6	11.2	11.3	0.18	12.7^c^	11.2^b^	10.4^a^	11.0^b^	0.17	11.6^b^	11.1^a^	0.13
	1							13.0^e^	11.8^cd^	10.3^a^	11.3^bc^	0.23			
	2							12.3^d^	10.6^ab^	10.5^a^	10.8^ab^				
		1	12.1^fg^	13.0^h^	12.4^gh^	13.2^h^	0.33								
		2	11.3^c-f^	11.8^efg^	11.5^def^	10.4^abc^									
		3	10.6^a-d^	10.3^ab^	9.9^a^	10.7^a-d^									
		4	10.7^a-d^	11.4^def^	11.0^b-e^	10.8^a-d^									
GE (MJ/kg)			2.74	2.89	2.75	2.74	0.060	3.23^d^	2.76^c^	2.50^a^	2.63^b^	0.049	2.86	2.69	0.042
		1	3.08^fgh^	3.35^h^	3.14^gh^	3.35^gh^	0.099								
		2	2.78^cde^	2.90^efg^	2.85^ef^	2.50^abc^									
		3	2.56^a-d^	2.49^ab^	2.38^a^	2.56^a-d^									
		4	2.55^a-d^	2.80^de^	2.65^b-e^	2.54^a-d^									
SCC (× 1000)			2.38	1.52	1.78	1.29	0.314	1.83	1.89	1.47	1.78	0.262	1.85	1.63	0.221
Yield															
Milk (g/day)			2224	2594	2390	2446	172.8	2404^b^	2637^c^	2460^b^	2153^a^	90.9	2059^a^	2767^b^	121.6
		1						1965^a^	2190^b^	2122^b^	1960^a^	127.8			
		2						2844^c^	3083^d^	2797^c^	2346^b^				
Fat (g/day)			84	106	91	91	6.6	113^d^	101^c^	83^b^	73^a^	3.9	84^a^	102^b^	4.6
	1							98^cd^	91^bc^	73^a^	73^a^	5.5			
	2							128^e^	111^d^	94^c^	76^ab^				
		1	99^def^	129f.	110^ef^	114f.	7.8								
		2	90^b-e^	117f.	104^def^	93^cde^									
		3	80^abc^	90^b-e^	76^abc^	86^a-d^									
		4	65^a^	88^bcd^	73^abc^	72^ab^									
Protein (g/day)			55	64	58	60	3.9	68^c^	62^b^	54^a^	53^a^	2.2	52^a^	66^b^	2.7
	1							58^b^	55^b^	46^a^	49^a^	3.1			
	2							78^d^	69^c^	63^b^	57^b^				
Lactose (g/day)			92	110	100	104	5.5	104^b^	110^c^	101^b^	92^a^	4.0	88^a^	116^b^	5.1
	1							89^a^	95^b^	85^a^	85^a^	5.6			
	2							121^cd^	124^d^	117^c^	100^b^				
TS (g/day)			246	300	266	273	18.5	301^c^	291^c^	256^b^	236^a^	10.3	238^a^	304^b^	13.0
	1							256^bc^	258^bc^	218^a^	221^a^	14.5			
	2							346^e^	325^d^	294^c^	251^ab^				
GE (MJ/day)			6.02	7.42	6.51	6.63	0.453	7.65^d^	7.16^c^	6.15^b^	5.62^a^	0.256	5.89^a^	7.40^b^	0.319
	1							6.54^bc^	6.39^bc^	5.28^a^	5.35^a^	0.360			
	2							8.75^e^	7.93^d^	7.02^c^	5.90^ab^				
GE:DMI (MJ/kg)			2.73	3.23	2.92	3.09	0.203	3.94^c^	3.02^b^	2.52^a^	2.48^a^	0.124	2.89	3.09	0.143

^1^GS = gestation supplement treatment; LD = lactation diet treatment; Mod = Moderate; PY = parity (1 or 2 =  ≥ 2); PD = period.

^2^TS = total solids; GE = gross energy (MJ/kg); SCC = log somatic cell count; DMI = dry matter intake (kg/day).

^a,b,c,d,e,f,g,h^Means within a grouping without a common superscript letter differ (*P* < 0.05).

*Mean values of main effects are presented when there were no significant interaction effects (*P* > 0.05) involving the main effects. Otherwise, data are presented showing interaction effect (*P* < 0.05) or significant main effects (*P* < 0.05).

Whole milk yield was not affected by GS or LD (*P* > 0.05), ranked period 2 > 1 and 3 > 4 (*P* < 0.05), and was greater for multiparous than for primiparous (*P* < 0.001; Tables 6, 7). But, there was an interaction between period and parity (*P* < 0.001), which was due to greater values for primiparous animals in periods 2 and 3 versus 1 and 4 (*P* < 0.05) and a ranking for multiparous animals (*P* < 0.05) the same as for overall period means. There were no effects of GS, LD, or GS × LD on yield of any milk component (*P* > 0.05). There was only one significant GS × LD × period interaction (*P* = 0.040), which was in milk fat yield. Yields of all constituents, as expected, were greater for multiparous versus primiparous animals (*P* < 0.05), and there were significant overall period effects as well (*P* < 0.05). Yields generally decreased with advancing period, and in most cases the magnitude of change was greater for multiparous than for primiparous animals. Relatedly, period main effect means for milk energy yield ranked (*P* < 0.05) period 1 > 2 > 3 > 4. Milk energy yield for multiparous goats differed among periods in the same manner (*P* < 0.05), but for primiparous goats means for periods 1 and 2 were greater than for periods 3 and 4 (*P* < 0.05). The ratio of milk energy to DM intake was not influenced by GS, LD, or parity (*P* > 0.05) and ranked (*P* < 0.05) period 1 > 2 > 3 and 4. However, the *P* value for LD was 0.100, with the ratio tending to be greater for High LD versus Moderate LD.

### Calorimetry measures and HE in the four periods

Measures during the two times (periods 2 and 3) animals were in the calorimetry system are addressed in Tables 8, 9, along with HR during each of the four periods and HE based on HR and the ratio of HE to HR determined in the calorimetry periods. There were no effects of GS or LD on any calorimetry measure (*P* > 0.05), but as for many other variables there were numerous differences between parities and among periods, as well as interactions (*P* < 0.05). For HR and HE determined in each period, values were greater for High LD than for Moderate LD (*P* < 0.05). There were three-way interactions (*P* < 0.05) in HR and HE relative to BW^0.75^ involving GS, parity, and period. With Moderate GS, these values were usually greater in period 1 than other periods for multiparous, but not for primiparous goats. Heat energy in MJ/day was greater for multiparous versus primiparous animals (*P* < 0.05) both during calorimetry measurements and all periods, although HE in kJ/kg BW^0.75^ tended to be greater during calorimetry measures (*P* = 0.051) but not in all periods based on HR (*P* > 0.05).

**Table 8:** *P* values for effects of gestation supplement treatment, lactation diet, and parity on calorimetry measures and predicted heat energy of Alpine goats during four 4-wk periods.

Source of variation^2^	Calorimetry measures^1^						HR and HE based on HR		
	RQ	HR	MET	HEMJ	HEMBW	HEHR	HR	HEMJ	HEMBW
GS	0.273	0.843	0.147	0.652	0.333	0.317	0.405	0.279	0.131
LD	0.816	0.350	0.465	0.358	0.119	0.247	0.026	0.020	0.003
GS*LD	0.821	0.900	0.103	0.090	0.501	0.680	0.985	0.150	0.659
PY	0.041	0.183	0.990	0.001	0.051	0.210	0.841	0.007	0.441
GS*PY	0.245	0.316	0.245	0.529	0.250	0.014	0.868	0.340	0.131
LD*PY	0.877	0.298	0.496	0.946	0.652	0.155	0.330	0.263	0.103
GS*LD*PY	0.684	0.335	0.924	0.388	0.189	0.413	0.348	0.492	0.369
PD	< 0.001	< 0.001	0.005	< 0.001	< 0.001	0.085	0.053	0.011	0.059
GS*PD	0.619	0.940	0.990	0.484	0.978	0.405	0.160	0.149	0.188
LD*PD	0.569	0.099	0.592	0.793	0.616	0.284	0.653	0.692	0.591
GS*LD*PD	0.199	0.387	0.308	0.982	0.989	0.593	0.853	0.858	0.786
PY*PD	0.136	0.007	0.086	< 0.001	0.002	0.719	0.255	0.077	0.172
GS*PY*PD	0.618	0.803	0.188	0.365	0.351	0.632	0.042	0.058	0.037
LD*PY*PD	0.680	0.286	0.259	0.057	0.007	0.178	0.710	0.766	0.799
GS*LD*PY*PD	0.991	0.477	0.582	0.357	0.539	0.888	0.195	0.269	0.223

^1^RQ = respiratory quotient; HR = heart rate (beats/min); MET = methane energy, MJ/day; HEMJ = heat energy, MJ/day; HEMBW = heat energy, kJ/kg BW^0.75^; HEHR = heat energy:heart rate, kJ/kg BW^0.75^:beats/min.

^2^GS = gestation supplement treatment; LD = lactation diet treatment; PY = parity (1 or 2 =  ≥ 2); PD = period.

**Table 9:** Effects of gestation supplement treatment, lactation diet quality, and parity on calorimetry measures and predicted heat energy of Alpine goats^1,*^.

Item^2^	PY	GS	LD	Moderate GS		High GS		SEM	Period				SEM	Parity		SEM
				Mod LD	High LD	Mod LD	High LD		1	2	3	4		1	2	
Calorimetry measures^3^																
RQ				1.07	1.07	1.05	1.05	0.014		1.03^a^	1.09^b^		0.009	1.08^b^	1.05^a^	0.010
HR				96.4	99.1	96.2	98.3	2.53		103.6^b^	91.4^a^		1.55	95.8	99.2	1.79
MET				1.59	1.90	1.62	1.50	0.128		1.47^a^	1.84^b^		0.090	1.65	1.65	0.090
HEMJ				14.5	15.9	15.2	14.7	0.54		15.8^b^	14.4^a^		0.31	14.1^a^	16.1^b^	0.39
	1									14.2^a^	14.0^a^		0.44			
	2									17.3^b^	14.8^a^					
HEMBW				702	760	694	717	25.6		749^b^	687^a^		14.2	693	744	18.1
	1									703^a^	682^a^	20.1				
	2									796^b^	693^a^					
	1		Mod							656^a^	677^ab^	28.5				
	2		Mod							795^cd^	664^a^					
	1		High							750^bcd^	687^ab^					
	2		High							796^d^	721^abc^					
HEHR				7.34	7.68	7.21	7.77	0.216		7.27	7.53		0.130	7.26	7.54	0.153
		Mod												7.65^b^	7.47^ab^	0.216
		High												6.88^a^	7.70^b^	
Measures in all periods																
HR				99.8	107	97.2	104	2.99	105	103	99.6	100	2.0	102	102	2.1
	1	Mod							99.8^abc^	102^abc^	106^cd^	103^abc^	4.1			
	2	Mod							112.0^d^	101^abc^	99.1^abc^	103^bc^				
	1	High							104^cd^	105^cd^	99.9^a^	100^abc^				
	2	High							104^cd^	105^cd^	99.2^abc^	94.8^ab^				
HE				15.3	17.6	15.5	16.1	0.59	16.8^b^	16.3^ab^	15.7^a^	15.8^a^	0.38	15.3^a^	16.9^b^	0.42
HEMBW				725	815	698	766	24.9	773	760	734	737	16.1	741	761	17.6
	1	Mod							755^bcd^	776^bcd^	805^cd^	782^bcd^	32.2			
	2	Mod							826^d^	740^abc^	724^abc^	754^bcd^				
	1	High							728^abc^	731^abc^	657^a^	697^ab^				
	2	High							784^bcd^	792^cd^	751^bcd^	715^ab^				

^1^GS = gestation supplement treatment; LD = lactation diet treatment; Mod = Moderate; PY = parity (1 or 2 =  ≥ 2).

^2^RQ = respiratory quotient; HR = heart rate (beats/min); MET = methane energy, MJ/day; HEMJ = heat energy, MJ/day; HEMBW = heat energy, kJ/kg BW^0.75^; HEHR = heat energy:heart rate, kJ/kg BW^0.75^:beats/min.

^3^Calorimetry measures were performed in periods 2 and 3.

^a,b,c,d^Means within a grouping without a common superscript letter differ (*P* < 0.05).

*Mean values of main effects are presented when there were no significant interaction effects (*P* > 0.05) involving the main effects. Otherwise, data are presented showing interaction effect (*P* < 0.05) or significant main effects (*P* < 0.05).

### BCS and BMI

There were no effects of GS or LD on BCS or any BMI (*P* > 0.05), although in agreement with many other measures there were numerous differences among periods and between parities (*P* < 0.05), but no interactions were significant (*P* > 0.05; Tables 10, 11). Body condition score was lower in periods 1 and 2 versus 3 and 4 and was greater for primiparous than for multiparous animals (*P* < 0.05). Conversely, BMI-WH, GMI-WP, and BMI-GP were greater for multiparous than for primiparous animals (*P* < 0.05). The BMI-GH, however, was lower for multiparous versus primiparous goats (*P* < 0.05).

**Table 10:** *P* values for effects of gestation supplement treatment, lactation diet, and parity on body condition score and body mass indexes of Alpine goats during four 4-wk periods.

Source of variation^2^	Variable^1^				
	BCS	BMI-WH	BMI-WP	BMI-GH	BM-GP
GS	0.823	0.704	0.778	0.463	0.530
LD	0.319	0.547	0.712	0.218	0.342
GS*LD	0.858	0.715	0.813	0.568	0.712
PY	< 0.001	0.002	< 0.001	0.010	0.005
GS*PY	0.399	0.890	0.137	0.961	0.947
LD*PY	0.475	0.691	0.540	0.996	0.685
GS*LD*PY	0.691	0.376	0.326	0.726	0.912
PD	< 0.042	0.001	0.001	0.067	0.383
GS*PD	0.806	0.117	0.811	0.262	0.299
LD*PD	0.820	0.765	0.869	0.248	0.163
GS*LD*PD	0.269	0.239	0.435	0.524	0.655
PY*PD	0.476	0.854	0.937	0.427	0.274
GS*PY*PD	0.546	0.808	0.581	0.649	0.562
LD*PY*PD	0.827	0.888	0.874	0.699	0.802
GS*LD*PY*PD	0.712	0.935	0.938	0.832	0.723

^1^BCS = body condition score; BMI = body mass index; Wither = height at withers; Hook = point of the shoulder to hook bone; Pin = point of the shoulder to pin bone; Heart = heart girth; BMI-WH = BW/(Wither × Hook) [g/cm^2^]; BMI-WP = BW/(Wither × Pin) [g/cm^2^]; BMI-GH = BW/(Heart × Hook) [g/cm^2^]; BMI-GP = BW/(Heart × Pin) [g/cm^2^].

^2^GS = gestation supplement treatment; LD = lactation diet treatment; PY = parity (1 or 2 =  ≥ 2); PD = period.

**Table 11:** Effects of gestation supplement treatment, lactation diet, and parity on body condition score and body mass indexes of Alpine goats during four 4-wk periods^1^.

Item^2^	Moderate GS		High GS		SEM	Period				SEM	Parity		SEM
	Mod LD	High LD	Mod LD	High LD		1	2	3	4		1	2	
BCS	2.89	2.94	2.89	2.96	0.057	2.85^a^	2.89^a^	2.97^b^	2.97^b^	0.034	2.98^b^	2.86^a^	0.040
BMI-WH	14.9	15.2	14.9	14.9	0.34	15.2^b^	15.0^b^	14.6^a^	15.0^b^	0.19	14.4^a^	15.5^b^	0.24
BMI-WP	11.8	11.9	11.8	11.8	0.24	12.1^c^	11.9^bc^	11.5^a^	11.7^ab^	0.14	11.4^a^	12.2^b^	0.17
BMI-GH	11.7	12.1	11.7	11.8	0.18	11.8	11.8	11.7	12.0	0.11	12.6^b^	12.1^a^	0.13
BMI-GP	9.27	9.44	9.24	9.31	0.127	9.35	9.26	9.28	9.37	0.078	9.13^a^	9.50^b^	0.089

^1^GS = gestation supplement treatment; LD = lactation diet treatment; Mod = Moderate; Parity = 1 or 2 (≥ 2).

^2^BCS = body condition score; BMI = body mass index; Wither = height at withers; Hook = point of the shoulder to hook bone; Pin = point of the shoulder to pin bone; Heart = heart girth; BMI-WH = BW / (Wither × Hook) [g/cm^2^]; BMI-WP = BW / (Wither × Pin) [g/cm^2^]; BMI-GH = BW / (Heart × Hook) [g/cm^2^]; BMI-GP = BW / (Heart × Pin) [g/cm^2^].

The correlation between BW and BCS and BMI were all significant (*P* < 0.05), with a lower value for BCS than for BMI (Table 12). There were no significant correlations involving ADG. Intake of DM in g/day was positively correlated with each BMI (*P* < 0.05), although the correlation between DM intake and BCS was negative but low (-0.12; *P* < 0.05). There were lesser numbers of significant correlations between BMI and DM intake relative to BW and, in particular, BW^0.75^, compared with DM intake in g/day. All correlations between ADG:DM intake and BMI were nonsignificant. In contrast to the negative correlation between BCS and NE_l_, there were low but significant and positive r between NE_l_ and the four BMI. There also was a negative relationship between BCS and NE_l_:DM intake (*P* < 0.001), but the relationship between NE_l_:DM intake and BMI were not significant (*P* > 0.05).

**Table 12:** Pearson correlation (r) between body condition score and body mass indexes and performance measures of Alpine goats.

Item^2^		Variable^1^							
		BW	ADG	DMI (g/day)	DMI (% BW)	DMI (g/kg BW^0.75^)	ADG:DMI	NE_l_	NE_l_:DMI
BCS	r	0.38	0.00	− 0.12	− 0.35	− 0.31	0.00	− 0.27	− 0.22
	*P*	< 0.001	0.957	0.033	< 0.001	< 0.001	0.957	< 0.001	< 0.001
BMI-WH	r	0.84	− 0.06	0.30	− 0.20	− 0.08	− 0.06	0.11	− 0.07
	*P*	< 0.001	0.329	< 0.001	< 0.001	0.144	0.293	0.048	0.244
BMI-WP	r	0.86	− 0.09	0.25	− 0.26	− 0.14	− 0.10	0.15	0.01
	*P*	< 0.001	0.092	< 0.001	< 0.001	0.012	0.066	0.007	0.830
BMI-GH	r	0.78	< 0.001	0.36	− 0.09	0.02	0.01	0.12	− 0.10
	*P*	< 0.001	0.963	< 0.001	0.100	0.743	0.907	0.038	0.066
BMI-GP	r	0.81	− 0.05	0.31	− 0.17	− 0.05	− 0.05	0.18	< 0.001
	*P*	< 0.001	0.340	< 0.001	0.003	0.335	0.381	0.002	1.000

^1^BW = body weight, kg; ADG = average daily gain, g; DMI = dry matter intake; NE_l_ = milk energy yield, MJ/day; NE_l_:DMI = MJ/kg DMI.

^2^BCS = body condition score, 1–5; BMI = body mass index; Wither = height at withers; Hook = point of the shoulder to hook bone; Pin = point of the shoulder to pin bone; Heart = heart girth; BMI-WH = BW/(Wither × Hook) [g/cm^2^]; BMI-WP = BW/(Wither × Pin) [g/cm^2^]; BMI-GH = BW/(Heart × Hook) [g/cm^2^]; BMI-GP = BW/(Heart × Pin) [g/cm^2^].

## Discussion

### Gestation

Even though GS supplements did not vary markedly in composition and all animals during the gestation phase had free access to alfalfa hay, the 5.5-kg difference in BW 11 days before kidding is appreciable. It reflects potential impact of even modest differences in feeding management practices during gestation. But it must be reiterated that the main difference in the nutritional plane resulting from the GS treatments was in the level of supplement feeding, 1.125% BW for the Moderate treatment and free-choice for ad libitum intake for the High treatment. It is notable that the difference in BCS at this time also was significant, although the slightly smaller magnitude may reflect the subjective nature of the measure and not necessarily reflect internal fat stores as noted in dairy cattle^5^. For example, the difference in BW divided by that in BCS of 0.18 yields a predicted change in BW of approximately 31 kg per unit BCS. This is obviously unrealistic and much greater than observed by Ngwa et al.^17^ in a more controlled and focused experiment specifically designed to assess such relationships. It is notable that the mean of the magnitude of differences in the four BMI between GS treatments was similar to that in BCS; however, that for the two BMI based on Wither were much greater than those based on Heart (i.e., approximately 7.8 versus 4.7%). There were significant differences in these measures between parities relative to ones for GS treatments.

Despite the considerable difference in pre-birth BW between doelings and does (i.e., 11.6 kg and 16.9% greater for does), BCS was only slightly but yet significantly greater (*P* < 0.05) for doelings than does. Although it can only be speculated upon at this point in time, this difference could involve a relatively greater proportion of body fat of does in internal versus subcutaneous storage sites compared with doelings as also suggested by results of Randby et al.^8^, with the latter having greater impact on BCS than the former. Perhaps in partial support of this postulate, BMI were greater for does than for doelings, again with the relative magnitude of difference greater for BMI based on Wither (BMI-WH and BMI-WP) than Heart (BMI-GH and BMI-GP).

Reproductive performances such as kid birth weight and litter size were similar between two GS, probably due to moderate differences in nutritional planes during gestation. Similar dam BW at birth for both GS treatments was the result of the much greater decrease in BW relative to the pre-birth for High GS than for Moderate GS. This cannot be explained by litter weight, which was not different between GS treatments (i.e., numerical 0.6 kg difference). Hence, the most plausible and logical explanation is greater mass of placentome tissues and fluids, notably the placenta, as depicted by the difference in change in total BW minus litter weight. A low nutritional plane in early to mid-gestation when maximum placental growth takes place has been reported to restrict placental mass^29,30^. For example, nutrient restriction in ewes at 30 to 80 days of pregnancy of 0.6 versus 2.25 times the maintenance energy requirement caused significantly lower fetal placental tissues (cotyledons) without any effect on maternal placental tissues (caruncles) or fetal weight^31^. Although not addressed in this study, it would be interesting and probably relevant to total production system profitability and sustainability to determine subsequent effects on postnatal growth and development of progeny such as health, immunity, early and later life growth, and reproductive performance^32,33^. Additionally, this suggests an importance of considering the life-long effects of such treatments rather than only considering variables characterizing dam conditions such as in this experiment of litter size and kid and litter birth weight, which, case-in-point, did not differ between GS treatments^32^. But there is cognizance that such long-term research programs are difficult to initiate and even are more challenging to maintain. The parity effect on birth BW and litter weight was also observed in other studies^34^.

Differences between GS treatments and parities in the magnitude of change in BW between kidding and the start of the lactation period were relatively small. Although differences were not large, BCS at the start of the lactation phase was slightly lower for each GS treatment and parity, probably at least partially reflecting a greater whole body fluid content before than after kidding with impact on the subjective BCS assessment. Relatedly, an interesting and seemingly important aspect of these results determined at different times, before, at, and soon after kidding, is value of measures before kidding that may not be predictable based on later ones. Moreover, it would be worthwhile to study the development of unique differences between nutritional planes throughout the mid- to late-gestation period.

### Lactation

#### Feed intake, BW, ADG, and blood constituents

Greater DM intake during the lactation phase for the Moderate than High GS treatment is in accordance with the difference in BW before kidding though not at that time or shortly thereafter. This is suggestive of a compensatory response for a difference in the nutritional plane during gestation. As alluded to before, this is notable since GS treatment did not significantly impact litter size or kid or litter weight, which may reflect the high priority of nutrient and energy use for pregnancy at the expense of other tissues of the dam. This difference in feed intake as well as that in pre-kidding BW indicates that conditions affected by the Moderate GS had an impact thereafter during lactation, which again very well could have also occurred in progeny development after birth.

Results of some other studies with cattle can be viewed in regard to how the magnitude and nature of differences in gestation diets can impact later feed intake. For example, Holstein cows subjected to a low plane of nutrition during late gestation and the close-up period displayed a tendency or had significantly greater feed intake in early lactation than the cows subjected to a greater plane of nutrition^35,36^. However, in similar studies no differences in feed intake between treatments were observed^16,37^ or intake was even greater for animals on a high plane of nutrition during gestation^14^. In the study of McNamara et al.^14^ with Holstein–Friesian cows, prepartum diets were grass silage and wheat straw in a 3:1 ratio, grass silage only, and grass silage plus 3 kg concentrate, with DM intake during the 4-wk pre-calving period of 7.4, 8.1, and 9.9 kg/day, respectively. During first 8 wk of lactation period, DM intake was greater for the highest versus lowest nutritional plane (e.g., 13.5 vs. 14.2 kg/day), with no difference for the diet of grass silage alone. Again, findings such as these may reflect impact of both the quantity and array of nutrients absorbed in gestation as well as the length of periods of dietary differences.

Though the GS × LD interaction was not significant for any DM intake expression, numerically values were greater for High LD than for Moderate LD with Moderate GS (i.e., differences of 246 g/day, 0.40% BW, and 12 g/kg BW^0.75^) converse to very similar values between LD with High GS (i.e., differences of only 39 g/day, 0.10% BW, and 2 g/kg BW^0.75^). These numerical differences suggest need for future studies with greater numbers of observations. Research with relatively greater differences in the gestation nutritional plane and(or) diet quality during lactation could be considered as well, but hopefully only with magnitudes relevant to practical production settings. Similarly, as implied above, in the study of Rabelo et al.^16^ there was not an interaction in feed intake during lactation of different dietary treatments during gestation and lactation.

The pattern of change in DM intake as period advanced is fairly typical of dairy goats as well as dairy cattle, with that in early lactation low relative to later periods^3,11^. Feed intake in period 4 was generally less than in periods 2 and 3, and it is presumed that values would have declined at least slightly had the lactation phase been longer. However, the pattern of change did vary somewhat between doelings and does, with a greater decline (8.4% versus 4.2%) in the latter segment (period 3 versus 4) of the phase for does, which corresponds with a greater decline in milk production of 16% versus 7.6% in this period. Research to identify factors responsible for this difference and to perhaps modify this pattern for higher intake in late lactation could be of interest to support milk production.

Results for BW and ADG suggest greater tissue mobilization by does versus doelings in early lactation (i.e., periods 1 and 2), although the *P* value for the parity × period interaction in ADG was 0.053. A relatively large amount of tissue mobilized by multiparous animals in the first period is in accordance with greatest milk energy yield at that time, similar to findings in some other studies^38–41^. Moreover, the GS treatment × period interaction reflects that High GS allowed for appreciably more tissue mobilization in period 1 than Moderate GS, which is in accordance with pre-birth BW results. It is notable that LD treatment did not impact BW or ADG presumably because of relatively small differences in ingredient and chemical composition and free-choice offering for ad libitum feed intake. Again, this probably relates to the nature of the LD diets being similar to that used in many dairy goat herds rather than having extreme characteristics that could have elicited many significant differences but ones of little practical value.

Relatively low glucose and high NEFA and BHB concentrations in blood are indicators of negative energy balance^42^. Differences among periods in blood glucose, with a higher level in period 2 versus 3 and 4, were likely due to greater milk lactose yield that is mostly synthesized from blood glucose^43^. Blood NEFA and BHB concentrations reflect adipose tissue lipolysis due to the energy demand for milk production and metabolism in the liver and peripheral tissues^42^. Factors responsible for the interaction of GS, LD, and period in blood BHB are unclear, and values did not seem reflective of differences in tissue mobilization or accretion. Blood lactate arises in ruminants from anaerobic glycolysis and absorption from the rumen. The cause(s) of the greater blood lactate concentration in period 2 than 3 and 4, especially for doelings, are also not readily apparent. This in part may relate to the lack of a sample in period 1.

#### Milk yield and composition

The lack of effect of GS and LD treatments and their interaction on raw milk yield and concentrations and yields of major constituents agrees with other results mentioned earlier, namely in feed intake and BW change. These findings reflect the ability of dairy goats to vary physiological conditions, such as in feed intake and compensatory effects of tissue mobilization or accretion, to maintain high lactation performance with dietary manipulations that can be considered of low to moderate magnitude. Likewise, there were no interactions between prepartum and postpartum diets in milk yield or levels of most constituents for Holstein cows fed diets similar in the concentration of net energy for lactation but differing in fermentability^15^. Conversely, in another study also with Holstein cows, prepartum and postpartum diets varying in starch concentration influenced milk yield and component levels^44^. However, there were significant effects of period and parity as well as numerous interactions as is common for dairy animals including goats^40,45,46^. For example, milk fat concentration decreased markedly as time advanced to period 3, with relatively greater change for High versus Moderate LD. The protein concentration was greater for doelings versus does, but this was mainly due to a difference only during period 2 probably because of greater milk yield by does in this period.

Period × parity interactions in milk component yields reflect greater magnitudes of change with advancing stage of lactation for does than for doelings. This at least in part should relate to continued growth and development of primiparous animals^47^, with relatively less energy and nutrients diverted for milk production compared with multiparous animals and, relatedly, less tissue mobilization for use in milk synthesis by the former animals. The magnitude of difference in milk energy yield between parities varied markedly with period, being greater in period 1 than in periods 2 and 3, with similar values in period 4. The ratio of milk energy yield to DM intake depicts greatest tissue mobilization in period 1, the least in periods 3 and 4, and an intermediate level in period 2. The lack of a difference in the ratio between doelings and does and a nonsignificant parity × period interaction could involve compensation of tissue mobilization by does and use of energy and nutrients for continued development of nonmaternal tissues by doelings. Lower milk production and a slower rate of decline in milk yield with advancing time for doelings versus does are consistent with other studies on sheep and goats^45,48,49^. The higher lactation persistency and flatter lactation curve are associated with greater rate of mammary gland epithelial cell proliferation, angiogenesis, and survival of primiparous goats, which continues for a relatively longer period of time compared with does^50^.

### Calorimetry measures and HE

The lower RQ in period 2 versus 3 and for does versus doelings were likely due to fat metabolism to support milk yield because fatty acid catabolism results in lower RQ, which is further substantiated by differences in BCS. Greater methane production in period 3 than in period 2 was not expected as feed intake in these periods was similar^51^. Therefore, physiological stages associated with differences in milk production performance and ruminal microbiota dynamics might have influenced methane production^52^.

Factors responsible for greater HE in both MJ/day and kJ/kg BW^0.75^ for the High LD than for the Moderate LD may be numerically greater DM intake and milk energy yield for High LD, as supported by other reports (e.g., Koong et al.^53^; Morris et al.^54^; Yan et al.^55^), with most of the differences attributable to the Moderate GS treatment. In accordance with greater milk production with associated metabolism to synthesize nutrients and DM intake by does versus doelings, HE was higher for does^54,56^.

The difference between LD in HE was not reflected in the ratio of milk energy to DM intake, with in fact a numerically greater value for the High LD. But, based on diet ingredient concentration and a lower level of NDF in the High LD than Moderate LD, the High LD would be expected to be somewhat more digestible than the Moderate LD. Lower HE in MJ/day in period 1 than 3 and 4, with an intermediate value for period 2, probably relates to period differences in milk yield and feed intake. Relatedly, the lack of a significant difference among period main effect means for HE in kJ/kg BW^0.75^ could involve changes in BW corresponding to tissue mobilization early in lactation and accretion later.

### BCS and BMI

The relatively small difference in BCS between periods is somewhat surprising given differences in milk energy yield and ADG, although BCS is a subjective measure as noted before. Nonetheless, lower BCS in the first two periods versus other periods relates to greater milk yields along with low DM intake. The ME requirement during the transition period increases two- to three-fold, resulting in mobilization of energy from fat depot^57^. Differences in BMI among periods were not substantial, and there was not one BMI for which values for periods 3 and 4 were greater than for periods 1 and 2 as was the case for BCS. Correlations between BCS and all BMI were highly significant, positive, and of low to moderate magnitude. Correlations between BCS and BMI based on Heart were lower than for BMI based on Wither. Findings of Liu et al.^20^ with yearling Alpine doelings were fairly similar, with highest r between BCS and BMI-WH and BMI-WP (0.35–0.40).

Significant correlation between BW and BMI were expected of course^58^. The lower correlation between BW and BCS may relate to subjectivity of the BCS measure and greater fat mobilization from internal sites relative to carcass and subcutaneous tissues^17,38,58,59^. The nonsignificant correlation between ADG and BCS as well as all BMI probably relates to the short 4-wk periods. The negative r between BCS and each DM intake expression could be reflective of animals with high feed intake having relatively high milk energy yield, perhaps with greater tissue mobilization and less accretion. This is in accordance with the negative correlation between BCS and milk energy yield and the ratio of milk energy yield to DM intake.

## Conclusions

Two gestation supplements fed at different rates before and during gestation elicited differences in BW of Alpine doelings and does before kidding but not litter size or kid and litter weights. A greater decline in dam BW from 11 days prior to and the day of birth for High GS than for Moderate GS suggests potential for subsequent progeny effects. Two lactation diets fed in early to mid-lactation had relatively minor effects on milk yield and constituent concentrations, without marked interactions involving GS treatment. Overall, without substantial differences in these nutritional planes in gestation and lactation, the dairy goats displayed a considerable capacity to alter physiological conditions such as feed intake, tissue mobilization and accretion for support of gestation and milk production. Future studies should consider factors such as effects on offspring development and productivity as well as future lactation performance of dams.

## Data Availability

All data generated or analyzed during this study are included in this published article.
